# *USP7* Regulates *TRPV1* Deubiquitination to Mediate Chondrocyte Ferroptosis and Alleviate Osteoarthritis

**DOI:** 10.1002/kjm2.70162

**Published:** 2026-01-09

**Authors:** Jun Li, Ding Chen, Wei‐Ye Zhong

**Affiliations:** ^1^ Department of Orthopedic Surgery The Second Xiangya Hospital and Central South University Changsha Hunan China

**Keywords:** chondrocyte ferroptosis, deubiquitination, osteoarthritis, TRPV1, USP7

## Abstract

Osteoarthritis (OA) is a common degenerative joint disease characterized by chondrocyte dysfunction. In this study, we explored the function and mechanism of ubiquitin‐specific protease 7 (USP7) in chondrocyte ferroptosis in OA. The USP7, TRPV1, collagen II, and GPX4 levels in knee joint tissue were detected using immunohistochemistry. Safranin O‐Fast Green staining detected histopathological changes in OA mice. RT‐qPCR and western blotting determined the expression of USP, TRPV1, collagen II, iNOS, MMP13, and MMP3. Primary mouse chondrocytes were treated with IL‐1β in vitro to simulate OA. Chondrocyte viability was assessed using the MTT assay. Immunofluorescence staining revealed an increase in reactive oxygen species levels. MDA and Fe^2+^ levels were measured using appropriate kits. The interaction between USP and TRPV1 was detected using co‐immunoprecipitation. An immunoprecipitation assay was used to evaluate TRPV1 ubiquitination. USP7 expression was downregulated in OA mice and IL‐1β‐stimulated chondrocytes. USP7 overexpression inhibited IL‐1β‐stimulated ferroptosis and extracellular matrix (ECM) degradation in chondrocytes. USP7, a deubiquitinating enzyme for TRPV1, enhanced TRPV1 stability Reinforced TRPV1 suppressed IL‐1β‐triggered chondrocyte ferroptosis and ECM degradation. Silencing TRPV1 reversed the effect of USP7 upregulation on IL‐1β‐induced chondrocyte ferroptosis and OA in OA mice. Therefore, USP7 protects chondrocytes from ferroptosis and ameliorates OA progression by deubiquitinating and upregulating TRPV1, thus providing a new therapeutic target for OA treatment.

**Abbreviations**
CHX: cycloheximide
Co‐IP: co‐immunoprecipitation
DMM: destabilized medial meniscus
DUBs: deubiquitinases
ECM: extracellular matrix
GPX4: glutathione peroxidase 4
IL‐1β: interleukin‐1beta
MDA: malondialdehyde
MTT: 3‐(4,5‐dimethylthiazol‐2‐yl)‐2,5‐diphenyltetrazolium bromide
OA: osteoarthritis
ROS: reactive oxygen species
SD: standard deviation
TRPV1: transient receptor potential vanilloid 1
USP: ubiquitin‐specific peptidase
USP7: ubiquitin‐specific protease 7

## 1 Introduction

Osteoarthritis (OA) is a prevalent degenerative joint disease that causes joint swelling, pain, and dysfunction, seriously affecting the quality of life. With an aging population and increase in obesity, the global prevalence and incidence of OA have increased, making it the most common joint disease [1]. The treatment of OA mainly includes symptomatic and surgical treatments such as joint replacement. However, traditional treatment methods do not effectively prevent the pathological progression of OA. Hence, a deeper understanding of the pathogenesis and treatment of OA is crucial. OA pathogenesis involves articular cartilage destruction, subchondral bone sclerosis, and osteophyte formation [2]. Chondrocytes, the only cell type in articular cartilage, participate in the production and degradation of the extracellular matrix (ECM). Interleukin‐1beta (IL‐1β), a widely recognized proinflammatory cytokine, is critical in OA development [3]. It simulates the inflammatory microenvironment in the pathological state of OA and induces phenotypic changes similar to those in OA in chondrocytes, such as ECM degradation and inflammatory response activation [4]. Therefore, it is widely used to establish in vitro OA models to investigate the cellular‐level pathogenesis and potential therapeutic targets of OA [5, 6]. With the progression of OA, inflammation, oxidative stress, and other factors in the joint tissue can induce excessive chondrocyte death, degrading the cartilage matrix, synergistically aggravating degeneration of articular cartilage in OA, and causing the loss of joint function [7, 8]. Different forms of chondrocyte death have been implicated in OA progression, with a particular focus on ferroptosis triggered by iron‐mediated lipid peroxidation [9].

Ferroptosis is cell death caused by iron‐dependent lipid peroxidation and the excessive production of reactive oxygen species (ROS). It differs from other cell death modes in terms of its morphology, biochemistry, and genetics. Excessive ROS production and lipid peroxidation can lead to chondrocyte damage, and ferroptosis is characterized by iron‐dependent lipid peroxide accumulation, suggesting that chondrocyte ferroptosis may participate in OA development [10]. Inhibition of chondrocyte ferroptosis is an effective method for delaying osteoarthritic cartilage degeneration [11]. Glutathione peroxidase 4 (GPX4), a lipid repair enzyme, exerts antiaging effects by directly reducing lipid peroxidation. Reduced GPX4 expression within OA cartilage results in increased oxidative stress and breakdown of the ECM [12]. These findings indicated the involvement of chondrocyte ferroptosis in the development of OA cartilage damage. However, the molecular features and possible treatment targets associated with ferroptosis in the OA cartilage remain largely unknown.

Deubiquitinating enzymes can hydrolyze the thioester bond between ubiquitin and proteins, remove ubiquitin molecules, reverse the ubiquitination process, protect the substrate from proteasome hydrolysis, and regulate the subcellular localization and activation of the substrate [13]. There are six families of deubiquitinases (DUBs), with the ubiquitin‐specific peptidase (USP) family being the largest [14]. Ubiquitin‐specific protease 7 (USP7) is a cysteine protease and a key member of the DUB family. USP7 regulates the activity and function of many cellular protein substrates, including tumor suppressor proteins, DNA repair proteins, immune response proteins, viral proteins, and epigenetic regulators, and is crucial in various diseases [15]. USP7 regulates inflammasome activation in macrophages [16], and its USP7 expression is downregulated in the knee joint cartilage of OA mice [17]. Thus, USP7 may regulate chondrocytes under inflammatory conditions. However, the precise mechanism through which USP7 functions in OA remains unclear.

Transient receptor potential vanilloid 1 (TRPV1), a transient receptor potential vanilloid receptor, can be activated by heat stimulation, acid, and inflammatory mediators [18]. Following activation, Ca^2+^ is preferentially allowed to pass through its ion channel, a large amount of extracellular Ca^2+^ flows into the cell and the intracellular Ca^2+^ concentration increases [19]. As secondary messengers, calcium ions can activate various signaling pathways that may be involved in the regulation of the cellular antioxidant defense system, iron metabolism, and lipid metabolism, thereby affecting ferroptosis. For example, calcium may affect the concentration of intracellular iron ions by activating certain kinases, promoting the synthesis of intracellular antioxidants, or regulating the activity of iron transporters, thereby inhibiting ferroptosis [20]. Furthermore, administration of a TRPV1 agonist via intra‐articular injection markedly alleviates pain in animal models of OA and in patients with OA [21, 22]. In an OA rat model, the intra‐articular injection of capsaicin, a specific TRPV1 agonist, notably reduced OA symptoms. Infiltrating OA synovial M1 macrophages highly express TRPV1, and its activation inhibits M1 macrophage polarization through the Ca^2+^/CaMKII/Nrf2 pathway [23]. Lv et al. showed that destabilized medial meniscus (DMM) induction notably reduced TRPV1 expression levels in damaged human OA and mouse cartilage. TRPV1 activation protects chondrocytes from ferroptosis and mitigates articular cartilage degeneration by upregulating GPX4 [24]. Another mechanosensitive ion channel, Piezo1, is associated with chondrocyte ferroptosis. Unlike TRPV1, when Piezo1 is activated by mechanical overload, the resulting calcium influx promotes ferroptosis. Conversely, the inhibition of this ion channel increased GPX4 expression and decreased ferroptosis [25]. In this study, we used bioinformatics to predict that USP7 is the only deubiquitinating enzyme of TRPV1. Therefore, USP7 may modulate TRPV1 deubiquitination and expression in chondrocytes during OA.

Based on these data, we speculated that USP7 may regulate TRPV1 deubiquitination. TRPV1 mediates Ca^2+^ influx to regulate ROS levels and promotes GPX4 expression, affecting chondrocyte ferroptosis and OA. This study provides a novel theoretical basis for OA treatment.

## 2 Materials and Methods

### 2.1 Construction of OA Animal Model

Eight‐week‐old male C57BL/6 mice (18–22 g) were obtained from the Shanghai SLAC Laboratory Animal Co. Ltd. (Shanghai, China). A DMM was used to induce OA according to established procedures [26]. In summary, mice were anesthetized via intraperitoneal injection of pentobarbital (40 mg/kg), and the medial meniscus on the right knee joint was surgically severed from the anterior tibial plateau. Thirty‐two mice were randomly divided into four groups (*n* = 8 per group): Sham, OA, OA + oe‐USP7, and OA + oe‐USP7 + sh‐TRPV1. In the sham group, mice underwent only skin and muscle incisions. One week after the surgical procedure, mice were intraarticularly injected with 5 μL (1 × 10^9^ pfu) of Ad‐oe‐USP7 or Ad‐sh‐TRPV1 (GenePharma, Shanghai, China). After an 8‐week period, the mice were euthanized, and their knees were harvested and fixed in 4% paraformaldehyde for further investigation. All animal experiments were approved by the Animal Care and Use Committee of the Second Xiangya Hospital and Central South University (animal ethical clearance number: 20251042).

### 2.2 Immunohistochemistry

After paraffin dehydration and clearing, knee joint tissue sections underwent antigen retrieval and blocking. Primary antibodies against USP7 (PA5‐34911, Invitrogen, Carlsbad, CA, USA), collagen II (PA5‐99159, Invitrogen), TRPV1 (75‐254, Invitrogen), and GPX4 (MA5‐32827, Invitrogen) were then applied, followed by incubation with a horseradish peroxidase‐conjugated secondary antibody. Immunohistochemical staining was visualized using 3,3′‐diaminobenzidine, and images were captured utilizing a microscope (Olympus, Tokyo, Japan).

### 2.3 Western Blotting

Total protein was extracted from tissues and chondrocytes using RIPA buffer (Beyotime, Shanghai, China). The protein concentration was determined using a BCA assay kit (Beyotime). Protein samples were separated on 10% SDS polyacrylamide gels and transferred to PVDF membranes (Millipore, Bedford, MA, USA). Next, membranes were blocked using 5% milk and then exposed to primary antibodies against USP7 (PA5‐34911, 1:1000, Invitrogen), TRPV1 (75‐254, 1:1000, Invitrogen), collagen II (PA5‐99159, 1:1000, Invitrogen), iNOS (PA1‐036, 1:1000, Invitrogen), MMP13 (MA5‐14238, 1:1000, Invitrogen), MMP3 (MA5‐42477, 1:1000, Invitrogen), and GPX4 (MA5‐32827, 1:1000, Invitrogen) overnight at 4°C. After washing three times with TBST, the membranes were incubated with HRP‐conjugated secondary antibodies (#7074, 1:1000, Cell Signaling Technology, Danvers, MA, USA) for 1 h. Protein bands were detected using a chemiluminescence reagent (Beyotime). The intensity was quantified using ImageJ software (National Institutes of Health [NIH], Bethesda, MD, USA) and normalized for β‐actin expression.

### 2.4 RT‐qPCR

Total RNA was extracted using the TRIzol reagent (Beyotime). The Prime Script RT Reagent Kit (Takara, Dalian, China) was used for reverse transcription. Subsequently, cDNA was amplified via RT‐qPCR using a reaction mixture comprising SYBR Green PCR Master Mix (Solarbio, Beijing, China) and examined using an ABI 7500 Fast Real‐Time PCR system (Thermo Fisher Scientific, Waltham, MA, USA). Primer sequences were as follows: mouse USP7 F: 5′‐GCCCTTTGGCCTGTAAATGAG‐3′, R: 5′‐AGTCTGAGCAACCCCAACAAA‐3′; mouse TRPV1 F: 5′‐AGCGAGTTCAAAGACCCAGAG‐3′, R: 5′‐TCTGTCTTCCGGGCAATGTC‐3′; mouse collagen II F: 5′‐AACACTGCCAACGTCCAGAT‐3′, R: 5′‐CTGCAGCACGGTATAGGTGA‐3′; mouse iNOS F: 5′‐TGACCATCATGGACCACCAC‐3′, R: 5′‐ACCAGCCAAATCCAGTCTGC‐3′; mouse MMP13 F: 5′‐TCATACTACCATCCTGCGACTCTTG‐3′, R: 5′‐TGCCAGTCACCTCTAAGCCAAAG‐3′; mouse MMP3 F: 5′‐TTCTCCAGGATCTCTGAAGGAGAGG‐3′, R: 5′‐ATTTGGTGGGTACCACGAGGACATC‐3′. GAPDH F: 5′‐AGCCCAAGATGCCCTTCAGT‐3′, R: 5′‐CCGTGTTCCTACCCCCAATG‐3′. Relative gene levels were calculated using the 2^−∆∆Ct^ method by normalizing with GAPDH.

### 2.5 Mouse Chondrocyte Isolation and Culture

As described previously [26], chondrocytes were isolated from the knee cartilage of 5‐day‐old C57BL/6J mice sourced from Charles River Laboratories (Beijing, China). The animal experimental protocols were approved by the Experimental Animal Ethics Committee of the Second Xiangya Hospital and Central South University. To summarize, cartilage samples were fragmented into small sections and underwent digestion with 0.25% trypsin for 30 min followed by 0.25% collagenase II for 6 h. Primary chondrocytes were then suspended in DMEM/F12 medium (Procell, Wuhan, China) supplemented with 10% fetal bovine serum (Gibco, Billings, MT, USA) and 1% penicillin–streptomycin and cultivated in at 37°C with 5% CO_2_. The first‐ and second‐generation chondrocytes were used in this study. Primary chondrocytes were incubated with recombinant mouse IL‐1β (0, 2.5, 5, 10 ng/mL, Peprotech, Cranbury, NJ, USA) [5, 6] or Erastin (10 μg/mL, Selleck Chemicals, Houston, TX, USA) for 24 h.

### 2.6 Cell Transfection

shRNA targeting USP7 (sh‐USP7: GCCGAATTTAACAGAGAGAAT) and TRPV1 (sh‐TRPV1: GCCATGCTCAATCTGCACAAT) and the negative control were purchased from GenePharma. The overexpression plasmids for USP7 and TRPV1 (oe‐USP7, oe‐TRPV1) were acquired from HANBIO Biological Technology (Shanghai, China). Cell transfection was conducted utilizing Lipofectamine 3000 (Thermo Fisher Scientific) for 48 h following the manufacturer's instructions.

### 2.7 3‐(4,5‐Dimethylthiazol‐2‐Yl)‐2,5‐Diphenyltetrazolium Bromide (MTT) Assay

The cells were plated in 96‐well plates and subjected to various treatments. Each well received 10 μL of MTT reagent (0.5 mg/mL, Promega, Madison, WI, USA) followed by incubation at 37°C for 4 h. Next, the culture medium was aspirated, and 150 μL of dimethyl sulfoxide was added to each well, shaken, and allowed to dissolve for 15 min. Cell viability was determined by measuring the absorbance at 490 nm using a microplate reader (Thermo Fisher Scientific).

### 2.8 Detection of ROS

Intracellular ROS levels were evaluated using the fluorescent probe DCFH‐DA, following the manufacturer's guidelines. Initially, chondrocytes were seeded in six‐well plates (3 × 10^5^ cells/well). Subsequent to the treatment grouping, the chondrocytes underwent three washes with PBS and were then exposed to 10 μM of DCFH‐DA (MedChemExpress, Shanghai, China) at 37°C in the dark for 30 min. After rinsing with PBS, cells were imaged under a fluorescence microscope (Olympus) for imaging capture.

### 2.9 Determination of Intracellular Fe^2+^ Concentration

To assess intracellular ferrous iron levels, the iron assay kit (Abcam, Cambridge, UK) was employed. Cell samples were collected, rinsed with cold PBS, and homogenized in an iron assay buffer. Subsequently, a 5‐μL iron reducer was introduced into Standard wells, whereas 5 μL of Assay Buffer was added to each sample, mixed thoroughly, and incubated for 30 min. Finally, a 100‐μL iron probe was added, mixed well, and left to incubate for 1 h. Absorbance at 593 nm was recorded using a microplate reader (Thermo Fisher Scientific).

### 2.10 Measurement of Cellular Malondialdehyde (MDA)

Following the specified treatments, chondrocytes were lysed in RIPA buffer and centrifuged at 12,000 × g for 5 min. The relative MDA content was assessed using a lipid peroxidation MDA assay kit (Beyotime). The reaction of MDA with thiobarbituric acid produced an MDA‐TBA adduct, which was quantified fluorometrically (excitation wavelength, 532 nm; emission wavelength, 553 nm).

### 2.11 Ubiquitination Assay

Chondrocytes were harvested and lysed in immunoprecipitation buffer containing a mixture of protease inhibitors. Following centrifugation (12,000 × g) for 15 min at 4°C, the cell supernatant was obtained and incubated overnight at 4°C with the appropriate antibodies. Subsequently, the samples were treated with the Protein A/G Plus‐Agarose Immunoprecipitation Reagent (Santa Cruz Biotechnology, DBA, Milan, Italy) at 4°C for 6 h. The beads were washed thrice with immunoprecipitation buffer and boiled in SDS buffer for 10 min. Proteins subjected to immunoprecipitation were separated using western blotting. The ubiquitination status of TRPV1 within the immune complexes was evaluated using an anti‐HA antibody (ab9110, Abcam).

### 2.12 Co‐Immunoprecipitation (Co‐IP)

The collected chondrocytes were lysed in immunoprecipitation buffer containing a protease inhibitor mixture. Following centrifugation at 12,000 × g for 15 min at 4°C, the cell supernatant was gathered and subjected to incubation with suitable antibodies specific to Flag (MA1‐142, Invitrogen), USP7 (PA5‐34911, Invitrogen), or IgG (R5130, Sigma, St. Louis, MO, USA). Subsequently, protein A + G agarose beads (Beyotime) were introduced, and the mixture was incubated at 4°C for 4 h. Following immunoprecipitation, the immunoprecipitates were washed four times with lysis buffer. Subsequently, the complex was boiled in a protein‐loading buffer for 5 min. The supernatant obtained using centrifugation was examined using western blotting to identify the expression of interacting proteins.

### 2.13 Detection of Protein Stability

Cycloheximide (CHX, Sigma) was employed to assess the stability of the TRPV1 protein. Briefly, 200,000 chondrocytes were seeded and incubated overnight. Subsequently, the cell culture medium was enriched with 100 μg/mL of CHX. Cell lysates were collected at 0, 2, 4, 8, and 16 h for TRPV1 assessment using immunoblotting.

### 2.14 Safranin O‐Fast Green Staining

Following euthanasia of the mice, the right knee joints were removed, fixed in 4% paraformaldehyde for 24 h, decalcified using a 10% EDTA solution for 2 weeks, and embedded in paraffin wax. Samples were sliced into 5‐μm sections in sagittal orientation before staining with safranin O/fast green (Sigma). Images were captured using a microscope (Olympus), and the results were analyzed using ImageJ software (NIH).

### 2.15 Statistical Analysis

SPSS software (version 25.0, IBM Corporation, Armonk, NY, USA) was used for statistical analysis. Data were expressed as the mean ± standard deviation (SD). Student's *t*‐test was used to compare the mean values between the two groups. For comparisons between multiple groups, one‐way analysis of variance was performed. All experiments were repeated at least thrice. *p* < 0.05 was considered significant.

## 3 Results

### 3.1 Reduced USP7 Expression in IL‐1β‐Treated Chondrocytes and the OA Mouse Model

**FIGURE 1:**
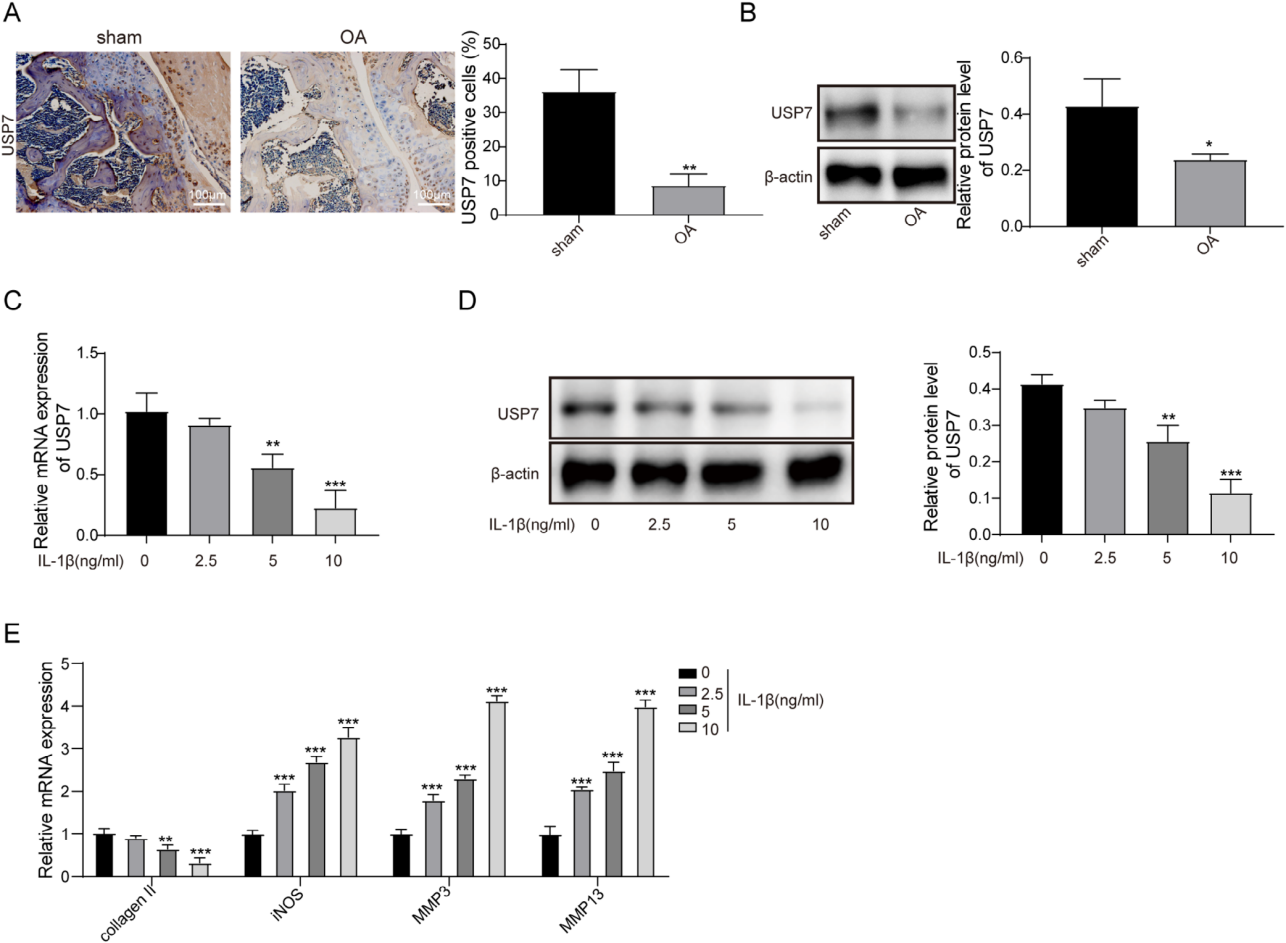
The expression of USP7 in IL‐1β‐induced chondrocyte model and OA animal model. (A) USP7 expression in knee joint tissue of Sham and OA mice was detected by immunohistochemistry. *n* = 5 mice per group. (B) Western blotting detected USP7 protein in knee joint tissue of Sham and OA mice. *n* = 5 mice per group. Next, primary mouse chondrocytes were isolated and treated with 0, 2.5, 5, 10 ng/mL IL‐1β to induce an OA cell model. (C, D) RT‐qPCR and Western blotting were used to assess USP7 expression. (E) Western blotting was used to determine levels of collagen II, iNOS, MMP3, and MMP13. Error bars stand for mean ± SD of at least triplicate experiments. *p < 0.05, **p < 0.01, ***p < 0.001.

To explore USP7 levels during OA progression in vivo, an OA mouse model was constructed. Immunohistochemistry and western blotting analyses showed that USP7 expression in the knee joint tissue of OA mice was significantly lower than that in Sham mice (Figure 1A,B). The primary mouse chondrocytes were isolated and treated with different IL‐1β concentrations to construct an OA cell model. RNA and protein levels of USP7 decreased with increasing IL‐1β concentration (Figure 1C,D). We explored the effect of IL‐1β on ECM degradation, and found that the RNA level of collagen II decreased with increasing IL‐1β concentration, whereas iNOS, MMP3, and MMP13 levels increased (Figure 1E). These results implied that USP7 was decreased in OA mice and IL‐1β‐stimulated chondrocytes.

### 3.2 USP7 Overexpression Inhibits Chondrocyte Ferroptosis and Alleviates ECM Degradation

**FIGURE 2:**
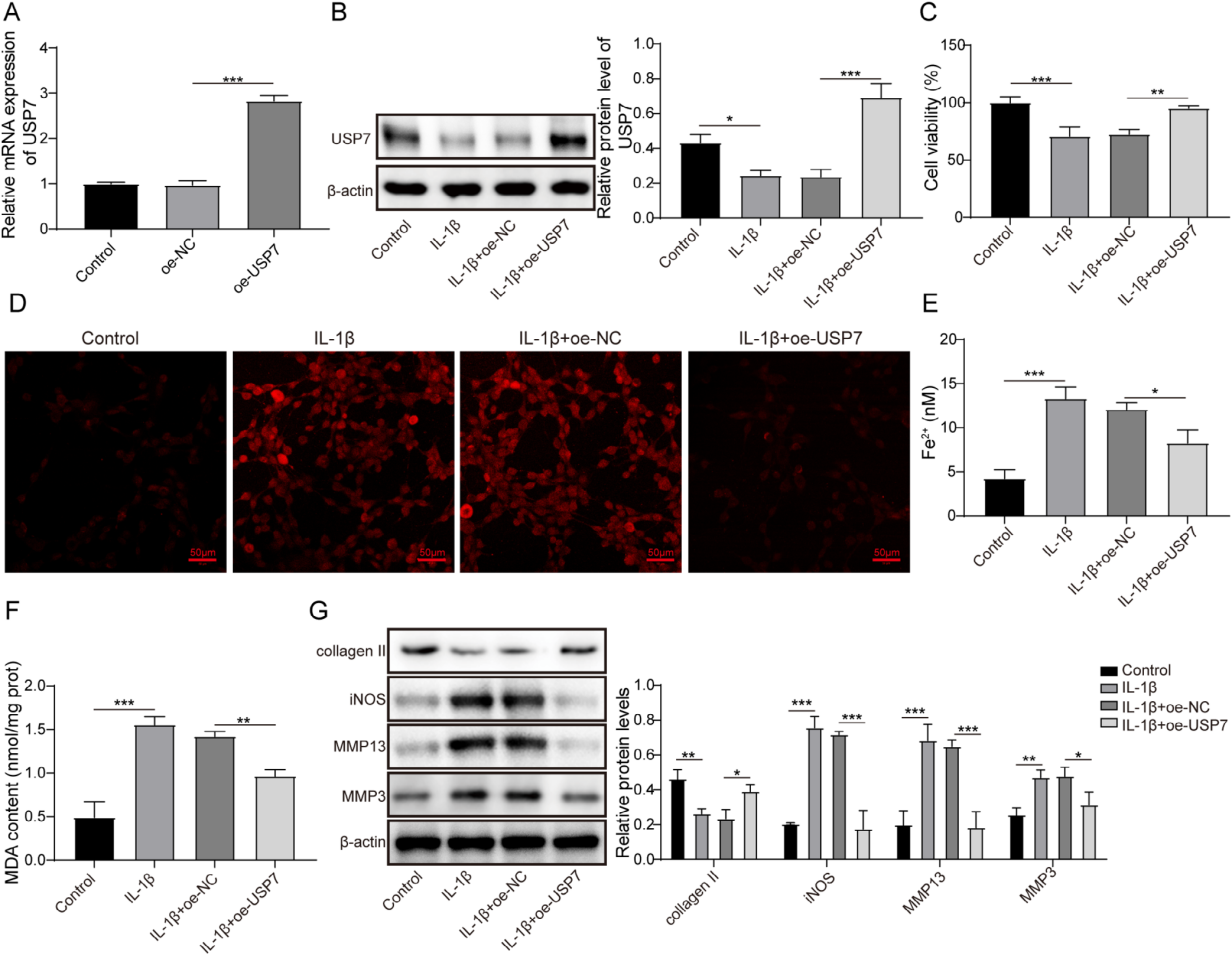
USP7 overexpression inhibited chondrocyte ferroptosis and alleviated ECM degradation. IL‐1β‐induced chondrocytes were transfected with oe‐NC or oe‐USP7. (A, B) RT‐qPCR and Western blotting were used to assess USP7 expression. (C) MTT assay evaluated cell viability. (D) Immunofluorescence was used to detect ROS levels. (E, F) Levels of iron content and MDA were measured by commercial kits. (G) Levels of collagen II, iNOS, MMP13, and MMP3 were detected by Western blotting. Data are the means ± SD for three independent experiments. *p < 0.05, **p < 0.01, ***p < 0.001.

To further evaluate the effect of USP7 on OA development, IL‐1β‐stimulated chondrocytes were transfected with either oe‐NC or oe‐USP7. USP7 overexpression promoted the RNA and protein expression levels of USP7 in chondrocytes (Figure 2A,B). MTT assay results showed that IL‐1β inhibited chondrocyte viability, whereas overexpression of USP7 reversed this phenomenon (Figure 2C). Immunofluorescence analysis revealed that IL‐1β significantly enhanced ROS levels, which were abolished by USP7 overexpression (Figure 2D). Furthermore, the IL‐1β‐induced increase in Fe^2+^ and MDA levels was reversed by oe‐USP7 (Figure 2E,F). Next, western blotting results showed that IL‐1β inhibited collagen II and promoted protein expression of iNOS, MMP13, and MMP3 in chondrocytes, whereas overexpression of USP7 reversed this effect (Figure 2G). These data indicate that USP7 upregulation can suppress IL‐1β‐triggered ferroptosis and degradation of ECM in chondrocytes.

### 3.3 USP7 Is a Deubiquitinating Enzyme of TRPV1 That Regulates TRPV1 Protein Stability

**FIGURE 3:**
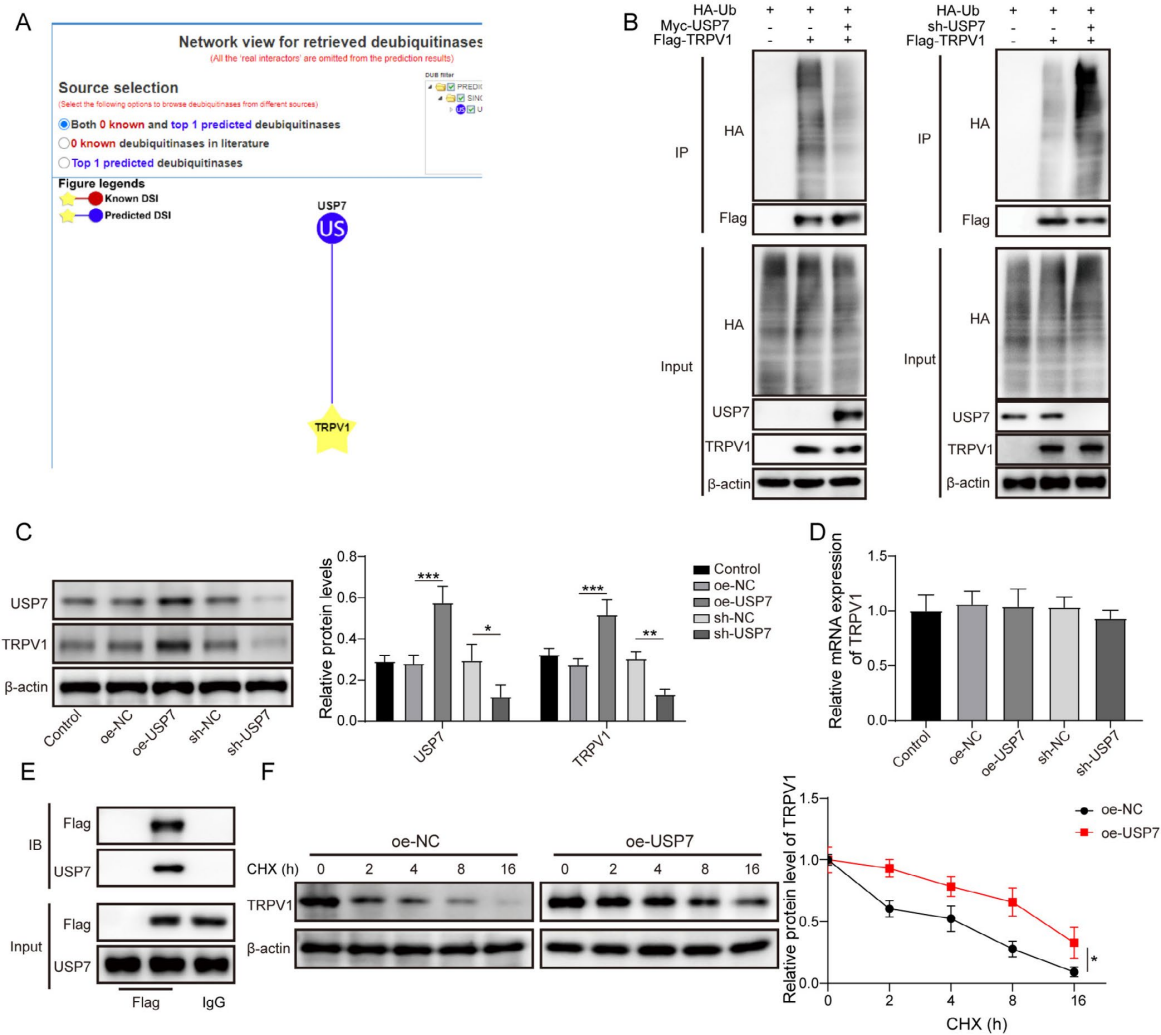
USP7 is a deubiquitinating enzyme of TRPV1 and regulated the stability of TRPV1 protein. (A) Bioinformatics website predicts that USP7 is a deubiquitinating enzyme of TRPV1. (B) Immunoprecipitation assay detected ubiquitination level of TRPV1 in chondrocytes. Next, chondrocytes were transfected with oe‐USP7 or sh‐USP7. (C, D) TRPV1 expression was detected by Western blotting and RT‐qPCR. (E) The interaction between USP7 and TRPV1 was detected by Co‐IP experiments. (F) Transfected chondrocytes were incubated with 100 μg/mL CHX for 0, 2, 4, 8, 16 h. Western blotting measured stability of TRPV1 protein. All experiments were repeated three times and error bars depict mean ± SD. *p < 0.05, **p < 0.01, ***p < 0.001.

In the present study, we investigated the downstream effectors of USP7. The Bioinformatics Website (http://ubibrowser.bio‐it.cn/ubibrowser_v3/) showed that USP7 is a deubiquitinating enzyme of TRPV1 (Figure 3A). Ubiquitination experiments showed that USP7 inhibited TRPV1 ubiquitination, whereas USP7 knockdown promoted TRPV1 ubiquitination (Figure 3B). Next, the chondrocytes were transfected with either oe‐USP7 or sh‐USP7. Western blotting analysis revealed that USP7 overexpression increased the protein expression levels of USP7 and TRPV1, whereas USP7 silencing had the opposite effect (Figure 3C). RT‐qPCR showed that neither knockdown nor overexpression of USP7 affected the RNA level of TRPV1 (Figure 3D). Co‐IP assays indicated that Flag‐TRPV1 and Myc‐USP7 plasmids were co‐transfected into chondrocytes, confirming the binding of exogenous TRPV1 to USP7 (Figure 3E). Subsequently, chondrocytes were treated with CHX, a protein synthesis inhibitor, revealing TRPV1 degradation, which decreased in CHX‐treated USP7‐overexpressing chondrocytes (Figure 3F). Taken together, these findings suggest that USP7 suppresses the ubiquitination and degradation of TRPV1 in chondrocytes.

### 3.4 Overexpression of TRPV1 Alleviates IL‐1β‐Triggered Chondrocyte Ferroptosis via GPX4

**FIGURE 4:**
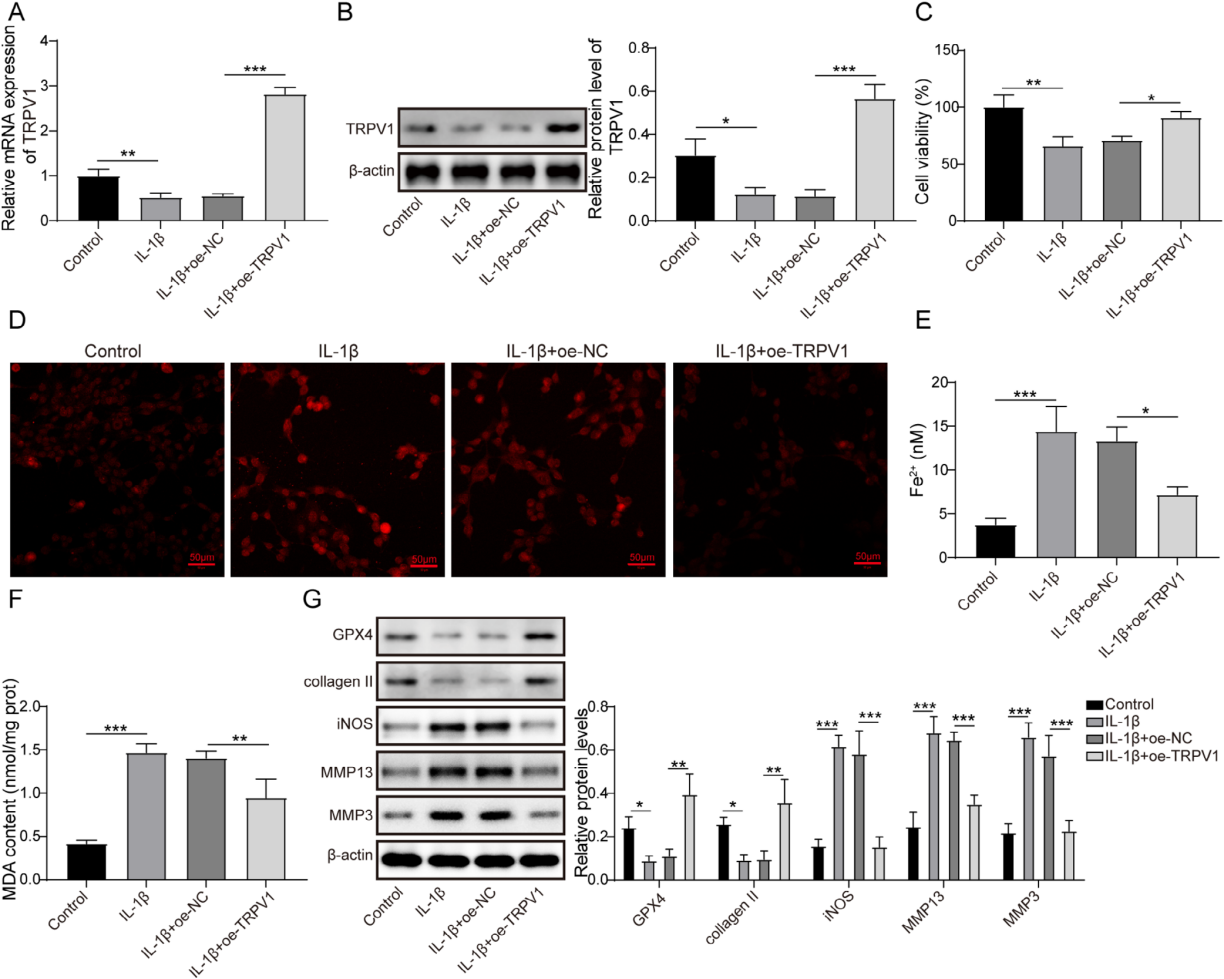
Overexpression of TRPV1 alleviated IL‐1β‐induced chondrocyte ferroptosis through GPX4. Chondrocytes were transfected with oe‐NC or oe‐TRPV1, and then treated with IL‐1β. (A, B) RT‐qPCR and Western blotting were used to assess TRPV1 expression. (C) MTT assay evaluated cell viability. (D) Immunofluorescence was used to detect ROS levels. (E, F) Levels of iron content and MDA were measured by commercial kits. (G) Levels of GPX4, collagen II, iNOS, MMP13, and MMP3 were detected by Western blotting. Data are the means ± SD for three independent experiments. *p < 0.05, **p < 0.01, ***p < 0.001.

To explore the effect of TRPV1 on OA development, IL‐1β‐exposed chondrocytes were transfected with oe‐NC or oe‐TRPV1. TRPV1 overexpression enhanced the RNA and protein levels of TRPV1 in chondrocytes (Figure 4A,B). MTT analysis demonstrated that IL‐1β inhibited chondrocyte viability, and overexpression of TRPV1 attenuated this effect (Figure 4C). Immunofluorescence results suggested that IL‐1β increased the chondrocyte levels of ROS, whereas overexpressed TRPV1 abrogated this upregulation (Figure 4D). Moreover, IL‐1β‐stimulated chondrocytes showed an increase in Fe^2+^ and MDA levels, whereas that increase was weakened by oe‐USP7 (Figure 4E,F). Furthermore, western blotting results showed that in chondrocytes, IL‐1β suppressed GPX4 and collagen II and enhanced the protein expression of iNOS, MMP13, and MMP3, which was reversed by TRPV1 overexpression (Figure 4G). Thus, our findings suggest that TRPV1 upregulation inhibits IL‐1β‐stimulated ferroptosis and ECM degradation in chondrocytes.

### 3.5 Knockdown of TRPV1 or a Ferroptosis Inducer Reverses the Effect of USP7 Overexpression in IL‐1β‐Induced Ferroptosis in Chondrocytes

**FIGURE 5:**
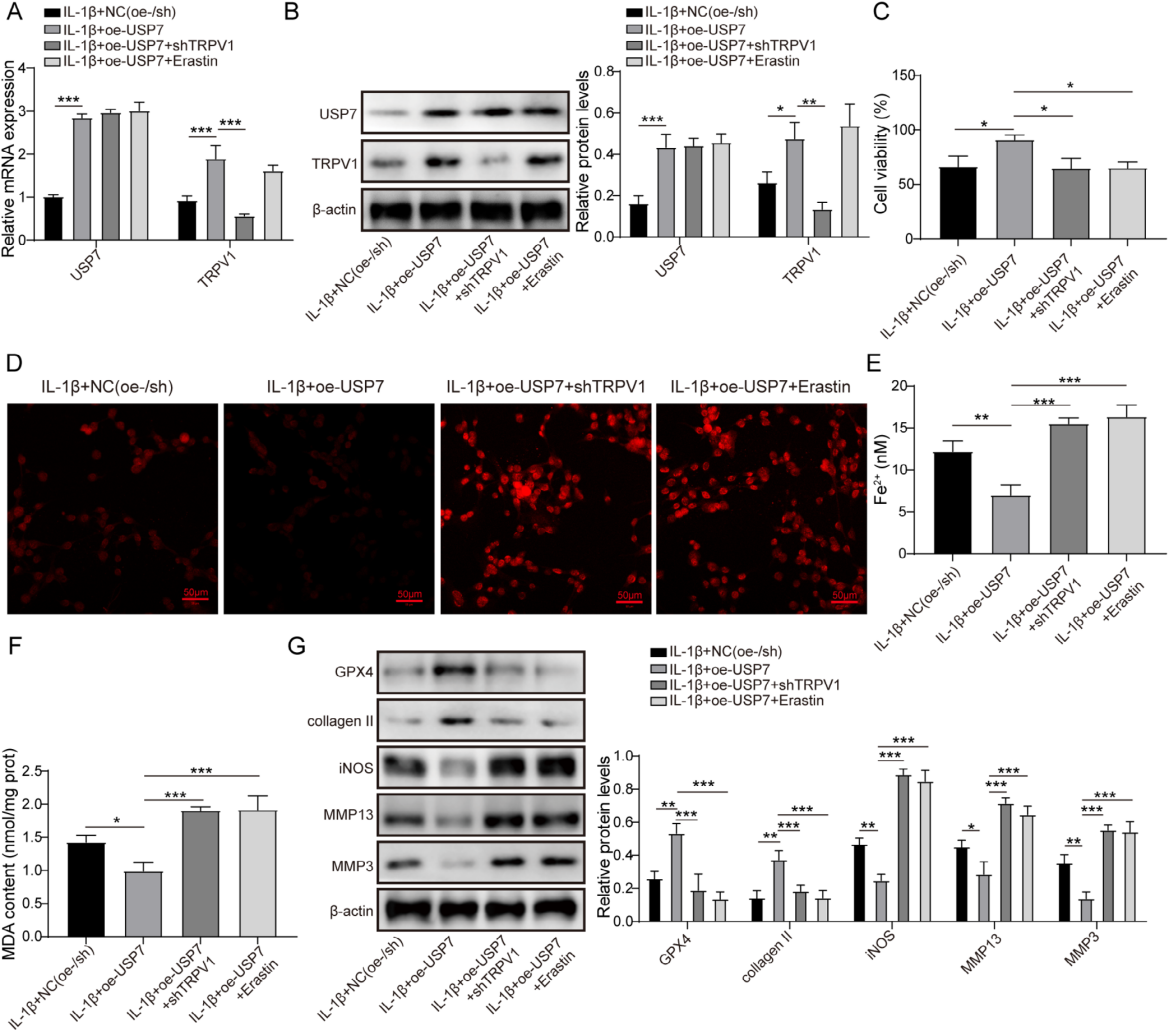
Knockdown of TRPV1 or ferroptosis inducer reversed the promoting effect of USP7 overexpression on IL‐1β‐induced ferroptosis in chondrocytes. Chondrocytes were transfected with oe‐USP7, sh‐TRPV1 and negative controls, and then treated with IL‐1β and Erastin. (A, B) RT‐qPCR and Western blotting were used to assess USP7 and TRPV1 expression. (C) MTT assay evaluated cell viability. (D) Immunofluorescence was used to detect ROS levels. (E, F) Levels of iron content and MDA were measured by commercial kits. (G) Levels of GPX4, collagen II, iNOS, MMP13, and MMP3 were detected by Western blotting. Values were expressed as mean ± SD of three separate determinations. *p < 0.05, **p < 0.01, ***p < 0.001.

We investigated the role of TRPV1 in USP7‐regulated chondrocyte ferroptosis in OA. Chondrocytes were transfected with oe‐USP7, sh‐TRPV1, and negative controls, and then treated with IL‐1β and Erastin. The data revealed that USP7 overexpression increased the RNA and protein levels of USP7 and TRPV1, whereas TRPV1 knockdown inhibited the RNA and protein levels of TRPV1 (Figure 5A,B). USP7 overexpression greatly enhanced chondrocyte viability induced by IL‐1β, which was then repressed by TRPV1 depletion or the addition of Erastin (Figure 5C). Additionally, reinforced USP7 markedly reduced the ROS level of chondrocytes induced by IL‐1β, which was partially improved by TRPV1 knockdown or Erastin administration (Figure 5D). The Fe^2+^ and MDA levels in chondrocytes induced by‐IL1β were suppressed by overexpressed USP7, followed by recovery by TRPV1 repression or Erastin treatment (Figure 5E,F). Additionally, USP7 overexpression promoted GPX4 and collagen II protein levels and inhibited iNOS, MMP13, and MMP3 in IL‐1β‐stimulated chondrocytes, whereas knockdown of TRPV1 or addition of Erastin reversed this phenomenon (Figure 5G). Taken together, TRPV1 silencing and ferroptosis inducers abolish the effect of USP7 upregulation on IL‐1β‐stimulated chondrocyte ferroptosis.

### 3.6 TRPV1 Knockdown Reverses the Effect of USP7 Overexpression on OA in OA Mice

**FIGURE 6:**
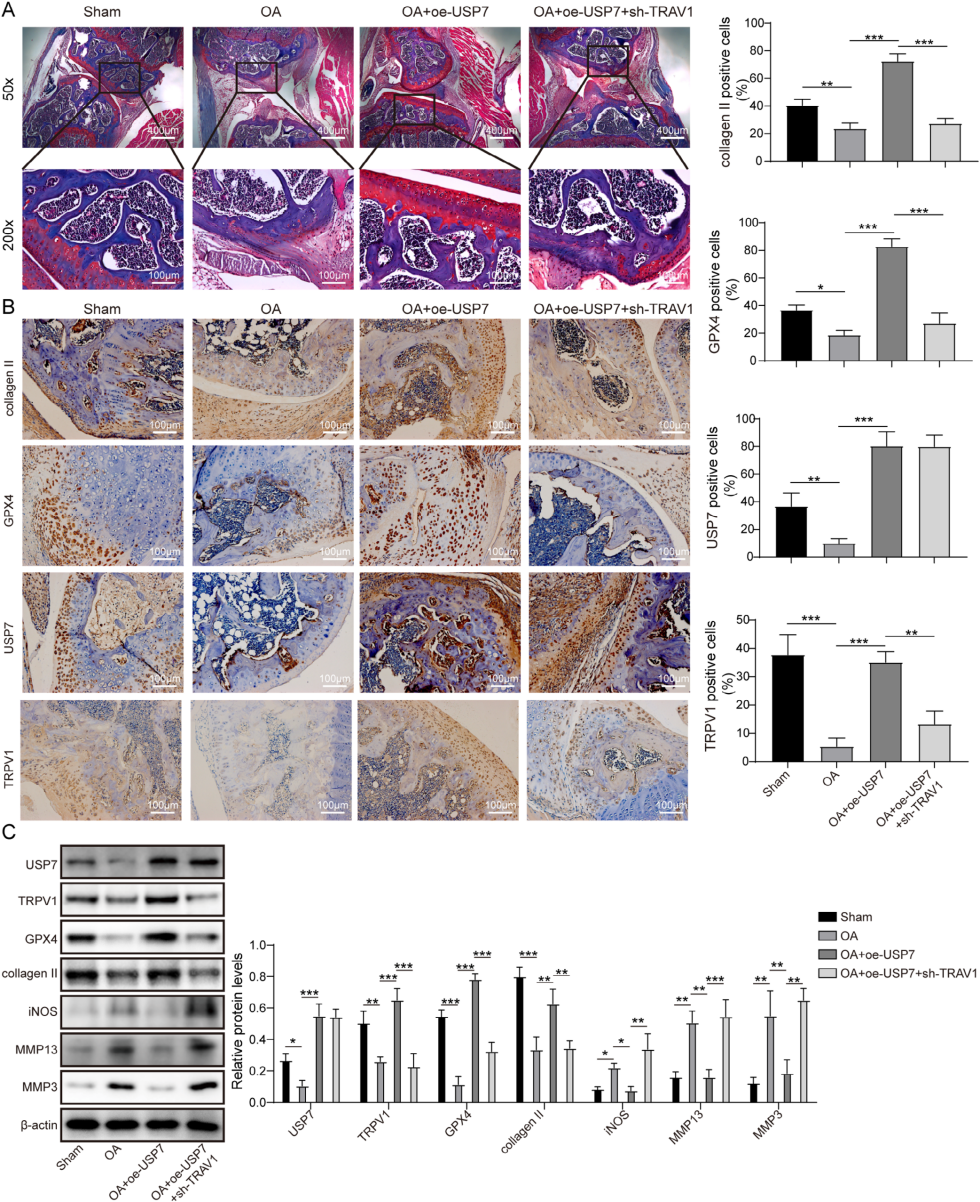
Knockdown of TRPV1 reversed the effect of USP7 overexpression on osteoarthritis in OA mice. The mice were divided into four groups: Sham group, OA group, OA + oe‐USP7 group, OA + oe‐USP7 + sh‐TRPV1 group. (A) Safranin O‐Fast Green staining of mouse knee joint. (B) Immunohistochemical staining of collagen II, GPX4, USP7, and TRPV1 in mouse knee joints. (C) Western blotting was used to detect levels of USP7, TRPV1, GPX4, collagen II, iNOS, MMP13, and MMP3. *n* = 5 mice per group. Data are the means ± SD. *p < 0.05, **p < 0.01, ***p < 0.001.

An OA mouse model was constructed to explore the role of the USP7/TRPV1 axis in OA progression in vivo. The mice were divided into the Sham, OA, OA + oe‐USP7, or OA + oe‐USP7 + sh‐TRPV1 group. Safranin O‐Fast Green staining of mouse knee joints indicated that OA mice exhibited hallmarks of OA; proteoglycan‐rich cartilage showed markedly reduced safranin O intensity, reflecting extensive proteoglycan loss; concurrent cartilage fibrillation and surface erosion further confirmed the disruption of ECM integrity and progressive cartilage degeneration. However, USP7 overexpression attenuated OA‐like performance, and TRPV1 knockdown further reversed the effect of oe‐USP7 (Figure 6A). Immunohistochemical staining revealed that the collagen II, USP7, TRPV1, and GPX4 levels in OA mice were decreased, whereas USP7 upregulation promoted collagen II, USP7, TRPV1, and GPX4 expression, and TRPV1 silencing further reduced TRPV1, GPX4, and collagen II protein levels (Figure 6B). In addition, western blotting demonstrated that the protein levels of collagen II, USP7, TRPV1, and GPX4 were decreased, and those of iNOS, MMP13, and MMP3 were increased in OA mice. Reinforced USP7 induced collagen II, USP7, TRPV1, and GPX4 expression and reduced iNOS, MMP13, and MMP3 expression. Depletion of TRPV1 further reduced TRPV1, GPX4, and collagen II levels, and elevated iNOS, MMP13, and MMP3 levels (Figure 6C). These results indicate that USP7 alleviates chondrocyte ferroptosis and ECM degradation by upregulating TRPV1 in vivo.

## 4 Discussion

OA is a frequently occurring disease that significantly affects the quality of life of individuals with degenerative joint diseases. OA is primarily caused by cartilage wear, secondary osteophytes, synovitis, and joint deformities. It is the main cause of disability and the need for knee arthroplasty in the elderly [27]. OA progression is linked to diverse types of chondrocyte death, inflammation, and oxidative stress [28, 29]. Ferroptosis, characterized by the excessive accumulation of iron‐dependent lipid ROS, represents a distinct form of cell death [30]. This study shows that USP7 is associated with chondrocyte ferroptosis in OA. In this study, we found that USP7 facilitated TRPV1 deubiquitination, thus inhibiting ferroptosis in chondrocytes. Therefore, USP7 is a promising therapeutic target for protecting chondrocytes against ferroptosis in OA.

Chondrocyte ferroptosis is a key event in OA progression [31]. In a DMM‐induced mouse model, the expression of ferroptosis‐related proteins in chondrocytes is abnormal, and inhibition of ferroptosis can significantly improve OA [11], indicating that targeted regulation of chondrocyte ferroptosis may be critical in treating OA. IL‐1β induces cellular inflammatory response and ferroptosis in various diseases including OA [32, 33]. Therefore, we used IL‐1β to treat chondrocytes to simulate OA model in vitro. IL‐1β promoted matrix destruction of chondrocytes and caused changes in chondrocyte ferroptosis, such as inhibition of GPX4; overexpression of iNOS, MMP3, and MMP13; accumulation of ROS; and increase in Fe^2+^ and MDA in chondrocytes, suggesting that IL‐1β induces chondrocyte ferroptosis ECM degradation.

The stability of several proteins is controlled by the ubiquitination and deubiquitination cycle [34]. As an important deubiquitinating enzyme, USP7 plays a role in regulating the inflammatory response, apoptosis, drug resistance, and other related pathophysiological mechanisms [35]. Under TNF‐α‐triggered inflammation, USP7 enhances chondrocyte proliferation while inhibiting apoptosis and inflammatory response [17]. Here, we found that USP7 expression was abnormally downregulated in OA mice and IL‐1β‐stimulated chondrocytes. In vitro, USP7 overexpression reduced chondrocyte ferroptosis and ECM degradation in IL‐1β‐treated chondrocytes. In rats, USP7 moderates HMOX‐1 through deubiquitination, reducing ferroptosis and improving spinal cord injury outcomes [36]. This is the first study to explore the molecular mechanisms by which USP7 modulates chondrocyte ferroptosis in OA. USP7 increases TRPV1 expression and stability through deubiquitination. The ion channel TRPV1 is involved in various processes and is linked to ferroptosis. TRPV1 suppresses ferroptosis and mitophagy to alleviate heart failure [37]. The gut microbiota metabolite capsiate enhances GPX4 expression and inhibits ferroptosis by activating TRPV1 during intestinal ischemia/reperfusion injury [38]. Interestingly, TRPV1 is an important antiferroptotic target in OA [39]. Activation of TRPV1 by capsaicin shields chondrocytes from ferroptosis in vitro, whereas pharmacological inhibition or silencing of TRPV1 via siRNA compromises this protective effect [23]. TRPV1 expression was notably decreased during OA progression, which was evident in human cartilage and an OA mouse model. The antiferroptotic effect of TRPV1 is due to its downstream target, which enhances GPX4 expression [24]. Notably, our results demonstrated that knockdown of TRPV1 or ferroptosis inducer counteracted the enhancement caused by USP7 overexpression on IL‐1β‐induced ferroptosis in chondrocytes. Further studies on the role of the USP7/TRPV1 axis in OA mice indicated that USP7 could mitigate chondrocyte ferroptosis and ECM degradation by increasing TRPV1 expression in vivo. Accordingly, these results suggest that USP7 plays a protective role in OA by activating TRPV1.

In conclusion, we report for the first time that USP7 inhibits chondrocyte ferroptosis and ECM degradation in OA by deubiquitinating and stabilizing TRPV1. Our findings provide a molecular basis for USP7 as a prognostic biomarker for precise and personalized treatment of OA. However, this study had several limitations. First, the effect of USP7 was assessed in cellular and animal models, necessitating further clinical investigations to confirm its efficacy in OA. In addition, exploring the association between USP7 and clinicopathological features of patients with OA is warranted. Moreover, the potential involvement of unidentified USP7 targets in the protective action against OA cannot be excluded. The functions of USP7 in OA in vivo should be verified by genetically knocked‐out mice. Further studies are warranted to determine whether USP7 plays a role in other cell types (such as synovial cells). However, further research is required to address these questions. Moreover, this study did not use pharmacological TRPV1 agonists (e.g., capsaicin) or antagonists (e.g., AMG‐9810) to test whether TRPV1 activation was required for the observed suppression of ferroptosis. Consequently, the use of TRPV1 as a therapeutic target in OA has not yet been substantiated by compound‐based evidence. Future studies should systematically evaluate TRPV1 modulation to determine the therapeutic potential of activating or inhibiting this channel both in vitro and in vivo.

## Ethics Statement

All procedures have been approved by the Animal Protection and Utilization Committee of the Second Xiangya Hospital and Central South University. The study was conducted in strict accordance with recommendations in the National Institutes of Health Guidelines for the Care and Use of laboratory animals. The study was conducted in accordance with the Declaration of Helsinki. All authors confirm that all methods are carried out in accordance with relevant guidelines and regulations.

## Conflicts of Interest

The authors declare no conflicts of interest.

## Data Availability

The data that support the findings of this study are available from the corresponding author upon reasonable request.
